# Molecular structural changes of Korat chicken meat as affected by packaging conditions during cold storage: Correlation to textural traits and sensory acceptance

**DOI:** 10.1016/j.psj.2025.106315

**Published:** 2025-12-19

**Authors:** Sylvia Indriani, Nattanan Srisakultiew, Soottawat Benjakul, Cynthia Andriani, Kanjana Thumanu, Passakorn Kingwascharapong, Samart Sai-ut, Jaksuma Pongsetkul

**Affiliations:** a School of Animal Technology and Innovation, Institute of Agricultural Technology, Suranaree University of Technology, Nakhon Ratchasima 30000, Thailand; b International Center of Excellence in Seafood Science and Innovation, Faculty of Agro-Industry, Prince of Songkla University, Hat Yai 90110, Thailand; c Department of Physics and Chemistry, Faculty of Science, University of Auckland, Auckland 1010, New Zealand; d Synchrotron Light Research Institute (Public Organization), Nakhon Ratchasima 30000, Thailand; e Department of Fishery Products, Faculty of Fisheries, Kasetsart University, Bangkok 10900, Thailand; f Department of Food Science, Faculty of Science, Burapha University, Chonburi 20131, Thailand

**Keywords:** Chicken meat, Meat texture, Protein degradation, Microstructure, Packaging

## Abstract

This study investigated microstructural and molecular changes in Korat chicken (**KC**) meat, a Thai hybrid known for its unique meat characteristics, under air packaging (**AP**), vacuum packaging (**VP**), and modified atmosphere packaging (**MAP**: 30% CO₂/70% N₂) and their effects on texture and acceptability during 12 days of cold storage. Changes in water- and salt-soluble proteins, insoluble collagen, as well as carbonyl and free thiol contents indicated that MAP and VP effectively slowed protein denaturation and mitigated oxidation during storage better thab AP (P < 0.05). Scanning electron microscopy (**SEM**) analysis confirmed less myofibrillar shrinkage and collagen degradation in VP and MAP. Both packaging methods also preserved better texture profiles, including shear force, hardness, and water-holding capacity (**WHC**), and changes in secondary protein structure, resulting in higher tenderness and juiciness scores throughout storage (P < 0.05). Principal component analysis **(PCA)** showed that α-helix were associated with Day 0 samples, whereas *β*-sheet and antiparallel *β*-sheet were associated with AP and VP/MAP samples, respectively, on Day 12. This suggested that O_2_ absence promoted the conversion of *α*-helix to antiparallel *β*-sheets, enhancing water retention and meat firmness, particularly during extended storage. Notably, on Day 12, MAP preserved WHC (70.16%) and tenderness score (6.62) better than VP (WHC: 68.02%; tenderness score: 5.91) (P < 0.05). This could be plausibly due to the role of CO₂ in shaping anaerobic bacterial growth and reducing physical stress caused by vacuum condition. Therefore, these findings offer insights into optimizing packaging for better texture preservation in KC meat, particularly under MAP.

## Graphical abstract

**Figure:**
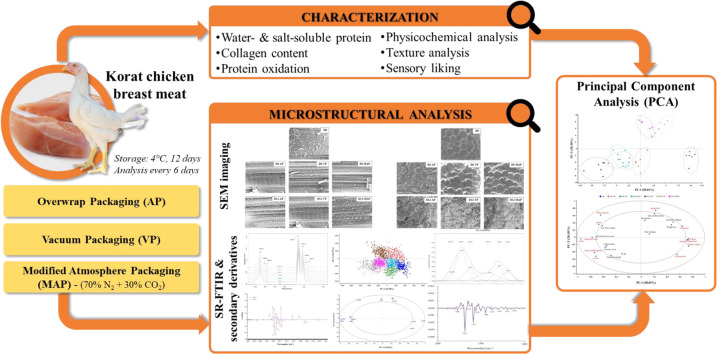

## Introduction

Chicken meat is a popular protein source due to its nutritious lean meat, lack of social or religious restrictions, and mild flavor, making it ideal for conventional and processed products (Kumar et al., 2022). Global demand for chicken meat has increased over the past 30 years, and by 2031, it is expected to make up 47% of all meat protein consumed, surpassing pork, lamb, and beef (FAO, 2023). Rural chicken farming in developing countries has become a practical way to meet this demand, especially through indigenous, native, and crossbreed chickens (Padhi, 2016; Panpipat et al., 2022). Korat chicken (**KC**), a new Thai hybrid bred from *Leung Hang Khao* sires and broiler dams developed in the Suranaree University of Technology (SUT) synthetic line, offers improved growth performance while maintaining the unique meat quality of native breeds (Katemala et al., 2022; Poompramun et al., 2022). KC meat, both fresh and cooked, exhibits distinct texture characteristics, such as being firmer and chewier due to its higher collagen content, shear force, and water-holding capacity (**WHC**), compared to broiler meat, as reported in previous studies (Katemala et al., 2021, 2022; Pongsetkul et al., 2024; Sangsawad et al., 2016). These traits cater to consumers who prefer the texture of native meat, highlighting KC’s strong potential in the marketplace as an important domestic livestock. Previous studies were mainly comparative research on particular distinctive meat qualities of KC with other chicken breeds as influenced by different rearing ages (Katemala et al., 2021), feeding patterns (Molee et al., 2022), and cooking conditions (Pongsetkul et al., 2024; Sangsawad et al., 2016). Recently, Indriani et al. (2025) reported distinct microbial dynamics in KC meat packaged under VP, MAP, and normal air conditions. These microbial shifts led to variations in spoilage development, driven by protein and lipid degradation, which in turn influenced flavor formation during storage for up to 12 days and ultimately resulted in differences in sensory acceptance. However, studies specifically monitoring changes in the textural quality of KC during storage, an essential quality attribute of this indigenous breed, remain limited.

In general, fresh chicken meat is highly perishable due to its high water activity and nutrient content, which promote rapid microbial growth, especially under conventional atmosphere packaging (**AP**) Marcinkowska-Lesiak et al. (2016). To slow spoilage, industry practices use vacuum packaging (**VP**) and modified atmosphere packaging (**MAP**) combined with cold storage (Cooksey, 2014; Hansen et al., 2023; Hauschild et al., 2021; Nauman et al., 2022). These methods reduce O_2_ levels, inhibiting the growth of aerobic spoilage bacteria, i.e., *Pseudomonas* spp. (Nauman et al., 2022). However, VP may promote the growth of anaerobic *Clostridium botulinum*, posing a potential safety risk if storage temperatures are not properly maintained (Peck et al., 2020). MAP has proven its effectiveness in ensuring chicken meat safety by significantly retarding the growth of *Campylobacter* spp., with a 30% CO_2_/70% N_2_ mixture performing best in preserving chicken meat during cold storage (Cha et al., 2023; Duqué et al., 2019). However, key considerations for MAP include the cost of gases, packaging materials, and machines used (Floros and Matsos, 2005). The alteration of chicken meat texture during storage, caused by packaging methods/conditions through biochemical reactions and metabolism of the meat and microorganisms, has been reported. For instance, MAP-packaged chicken meat retained a higher WHC and shear force during storage, thus prolonging shelf life and preserving sensory acceptability better than AP-packaged meat (Gurunathan et al., 2022). Marcinkowska-Lesiak et al. (2016) found that VP-packaged chicken breast meat tends to have higher drip loss, resulting in lower sensory acceptability during cold storage, particularly in terms of tenderness, stringiness, juiciness, and color. Changes in poultry meat texture, often linked to protein degradation and oxidation, are evaluated using metrics such as pH, cooking loss, drip loss, and WHC, alongside texture profile analysis (**TPA**) and electron microscopy (Carvalho et al., 2017; Guo et al., 2019; Pongsetkul et al., 2024). Despite previous findings, there is limited evidence on how different packaging conditions impacted the molecular microstructure and texture of KC breast meat during cold storage, which are key attributes reflecting the distinctive characteristics of this breed. This study, therefore, aimed to comprehensively monitor the microstructural and molecular changes in KC breast meat under various packaging conditions and evaluate their effects on texture and acceptability during storage. This research could provide valuable insights into optimizing packaging conditions to maintain KC meat’s desirable texture in the marketplace.

## Materials and methods

### Sample preparation

A total of 270 breast muscles (*Pectoralis major*) were obtained from 12-week-old Korat crossbred chickens (**KC**), comprising a mixed population of males and females, with an average carcass weight of 1.29 ± 0.41 kg, to represent commercially available chicken meat not segregated by sex. Samples were sourced from three commercial manufacturers in Nakhon Ratchasima, Thailand, in May 2024. All chickens were slaughtered at a commercial slaughterhouse, following standard commercial procedures. After slaughtering, the breasts were immediately collected and transported to the laboratory in an icebox within 1 h. Upon arrival, the breasts were deskinned, washed with distilled water, and cut into sizes of 120 ± 25 g each with a thickness of 1.5 ± 0.5 cm, then allowed to undergo 24 h postmortem aging in a 4°C-chiller, before undergoing various packaging treatments. Out of 270 breast muscles (90 pieces/lots), 30 pieces (10 pieces/lots) were analyzed prior to storage and packaging to referred to as ‘**Day 0**’ samples. The remaining 240 pieces (80 pieces/lots) were randomly assigned to three packaging treatments, with 60 pieces per treatment (20 pieces/lots), and further subdivided into two storage periods (30 pieces/treatment/storage day). For packaging treatment, the remaining breasts were individually placed on a polypropylene (**PP**) tray (18.5 × 12 × 3.5 cm^3^) and packed under three packaging conditions: (1) air packaging (**AP**), (2) vacuum packaging (**VP**), and (3) modified atmosphere packaging (**MAP**). For AP, the sample was packed in a regular atmospheric condition by overwrapping with a polyvinyl chloride (**PVC**) film. For VP and MAP, a nylon/polyethylene (PE) bag (20 × 30 cm^2^, 90 µm in thickness) with gas permeability of <15, 15, and 100 cm^3^/m^2^·day·atm for O_2_, N_2_, and CO_2_, respectively (at 75% relative humidity (**RH**), 25°C), and a water vapor permeability of <1 g/m^2^·day (at 85% RH, 25°C) was used. VP was performed using a vacuum-packing machine (FVC-II, Furukawa MFG Co. Ltd., Chiba, Japan) with a vacuum extent of 99.6%. MAP was performed using a Henkovac traysealer (type 1000, Tecnovac, Grassobbio, Italy) with a gas mixture of N_2_ = 70%, and CO_2_ = 30% at a gas/meat ratio of 3:1 (*v/w*). All samples were stored at 4°C for 12 days. For analysis, the samples (40 breasts) were each taken on Day 6 and Day 12 of storage.

### Structural and functional analysis

#### Protein solubility

Water- and salt-soluble proteins in the samples, which represented sarcoplasmic and myofibrillar proteins, respectively, were evaluated as per the method of Hultmann and Rustad (2002). Protein content was determined using the Biuret method, and bovine serum albumin (**BSA**) was used as the standard. The results were expressed as a percentage of the sample weight.

#### Total and insoluble collagen content

The collagen contents were measured as total and insoluble collagen according to the methods tailored by Zhu et al. (2023). Hydroxyproline was used as the standard to quantify the collagen content as a percentage.

#### Protein oxidation

Protein oxidation was assessed by measuring protein carbonyl and free thiol contents, according to the methods of Pongsetkul et al. (2022b). BSA was used as the standard, and the carbonyl content was expressed as nmol carbonyl/mg protein. L-Cysteine was used as the standard, and the free thiol content was expressed as nmol thiols/mg protein.

#### Microstructure

Scanning electron microscopy (**SEM**) was used to evaluate the microstructure of chicken breast samples. Briefly, the samples (1 × 2 × 1 cm^3^) were immersed in a 2.5% (*v/v*) glutaraldehyde solution (containing 0.2 M phosphate buffer (pH 7.2)) for 24 h at 4°C, then rinsed with distilled water. Subsequently, the sample was dehydrated in serially diluted ethanol solutions for 20 min. The samples were then cut (thickness = 3 mm) using a razor blade, placed on a bronze stub, and sputtered with 10 nm gold prior to the microstructure visualization using an SEM machine (S-3400N, Hitachi, Tokyo, Japan) at an operating voltage of 10 kV. Transverse and longitudinal slices were captured at magnifications of 500 × and 10,000 ×, respectively. The average fiber diameter and sarcomere length were then estimated based on 8 measurement areas from 5 micrographs (*n* = 50), using a morphometric approach as per the method of Pongsetkul et al. (2024).

#### Biomolecules and protein secondary structure analysis

Synchrotron Radiation-Based Fourier-Transform Infrared Spectroscopy (**SR-FTIR**) was employed to analyze biomolecules and protein secondary structures in the samples. For sample preparation, the samples (0.5 × 0.5 cm^2^) were embedded in an optimal cutting temperature (**OCT**) compound within aluminum foil blocks to prevent freezing damage, followed by flash-freezing in liquid nitrogen. The frozen tissue was sliced into 5 µm sections using a cryostat (Leica CM1950, Leica Biosystems Nussloch GmbH, Nussloch, Germany), mounted on a transparent BaF_2_ IR window (Crystran Ltd., Dorset, UK), and stored in a vacuum desiccator prior to SR-FTIR analysis. Each sample was replicated six times.

The biomolecule spectra of samples were obtained using an FTIR spectroscopy machine (Vertex 70) paired with an IR microscope (Hyperion 2000, Bruker Optics, Ettlingen, Germany), which was operated via OPUS software (version 7.8, Bruker Optics Ltd., Germany). Spectra were collected from 4000 to 400 cm^–1^ with a resolution of 4 cm^–1^ and 64 scans co-added. Each sample was replicated 30 times, totaling 180 spectra per sample. Thereafter, the spectra were processed using Unscrambler® X software (version 10.5, Camo Analytics, Oslo, Norway), employing a second derivative with a third-order polynomial and Savitzky-Golay smoothing (13 points) to minimize baseline variations. Vector normalization was applied, focusing on spectral ranges 3000–2800 cm^–1^ and 1800–900 cm^–1^ to enhance resolution and spectral characteristics. Principal component analysis (**PCA**) was used to assess variability in the data, explaining variance through the first few principal components (**PCs**). The intensity of protein secondary structures, annotated in the amide I and II regions (1700–1500 cm⁻¹), was calculated as percentages of *α*-helix, *β*-sheet, *β*-turn, and antiparallel conformations using Gaussian and Lorentzian functions in OPUS 7.8 software (Bruker Optics Ltd., Ettlingen, Germany).

### pH, WHC, weight and cooking loss

The sample (10 g) was homogenized with distilled water (100 mL) at 9,000 rpm for 2 min. Subsequently, the pH of the homogenate was measured using a digital pH meter (Sartorius, Göttingen, Germany). WHC was determined using the low-speed centrifugation method (1,000 rpm, 5 min, 4°C) described by Digre et al. (2011) and expressed as the percentage of water retained in the mince after centrifugation. After storage, the samples were patted dry with a paper towel and reweighed. The samples were cooked by boiling in water (90–100°C) until the internal temperature of the meat reached 71°C, held for 5 min, then instantly chilled in ice water, drained, and gently wiped with tissue paper to remove excess surface moisture before being re-weighed. Weight and cooking loss were determined and expressed as a percentage of the initial weight (*w/w*, wet basis), according to the method of Pongsetkul et al. (2024).

### Texture analysis

Cooked samples were used for texture analysis and prepared as mentioned above in Section 2.3. The cooked samples were cut into cubes (1 × 2 × 1 cm^3^) with muscle fibers aligned parallel to the longitudinal direction before being subjected to shear force and TPA using a Texture Analyzer (TA.XT.Plus, Stable Micro Systems, Surrey, UK). For shear force, a Warner-Bratzler blade with a test speed of 4 mm/s and a 50 kg load cell was set. For TPA, samples were compressed to 75% of their original height with a trigger force of 50 kg at a test speed of 2 mm/min. The hardness (N), chewiness (N*mm), springiness (mm), and cohesiveness (ratio) were calculated following the method of Panpipat et al. (2022).

### Sensory analysis

Sensory evaluation was conducted in accordance with the ethics regulations for human-subject research (**HSR**), including the Declaration of Helsinki, the Belmont Report, the CIOMS Guidelines, the International Conference on Harmonisation – Good Clinical Practice (ICH-GCP), and 45 CFR 46.101(b), as well as the guidelines recommended by Thailand’s National Research Council. All procedures described herein were supervised and approved by the Human Research Ethics Committee of Suranaree University of Technology (Document ID: EC-67-122). The scores for tenderness, juiciness, and overall texture likeness were evaluated by 50 untrained panelists (21–37 years old, 15 men, 35 women) using a nine-point hedonic scale (1 = dislike extremely, 9 = like extremely) to assess the texture acceptance of the samples. Signed consent forms from the participants in the sensory evaluation have been obtained regarding volunteer participation in this study. Prior to tasting, the samples were boiled (90–100°C) until the internal temperature of the meat reached 71°C and held for 5 min (without adding any salt or spices). Then, the cooked samples were cut into small cubes (1 × 2 × 1 cm^3^) and served with soy sauce on a white paper plate. The assessment took place in separate sensory evaluation booths illuminated with fluorescent white light. According to the ethic guideline, the total viable count (< 5 × 10^5^ CFU/g) and pathogens (*Staphylococcus aureus* (< 1 × 10^2^ CFU/g), *Bacillus cereus* (< 1 × 10^3^ CFU/g), *Salmonella* spp. (absence in 25 g), and *Listeria monocytogenes* (absence in 25 g)) were monitored in all samples prior to sensory evaluation and were found to be within the permissible limits for food safety.

### Statistical analysis

All measurements were performed in triplicate (*n* = 3) using samples from three independent lots, and results are reported as mean ± standard deviation (SD). Statistical analysis was conducted using two-way analysis of variance (**ANOVA**). An independent *t*-test was used to compare the means between two samples. Tukey’s test was used to determine statistical differences in all data at a significance level of 95% using SPSS statistical software (version 25.0, SPSS Inc., Chicago, Illinois, USA). PCA was employed to assess the relationship among all textural traits and sensory scores of the samples using the Unscrambler® X multivariate data analysis software (version 10.5, Camo Analytics, Oslo, Norway).

## Results and discussion

### Molecular structural changes of KC meat during storage

#### Water- and salt-soluble protein

**Fig. 1:**
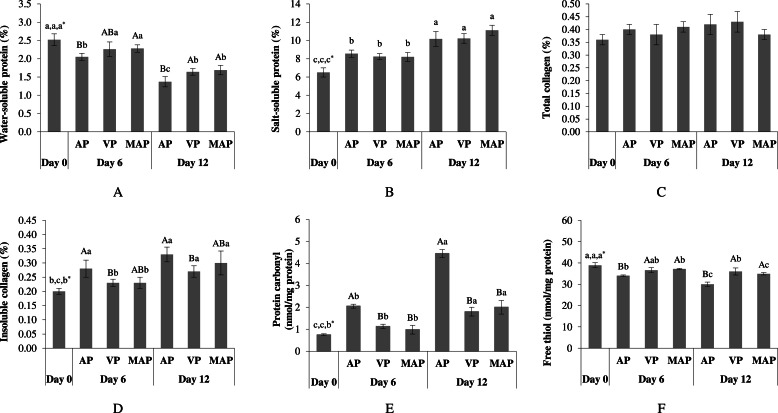
Water-soluble protein (A), salt-soluble protein (B), total collagen (C), insoluble collagen (D), protein carbonyl (E), and free thiol (F) of KC meat stored under various packaging conditions at 4°C for 12 days. Different uppercase letters on the bars indicate significant differences caused by different packaging conditions at the same storage time (P < 0.05). Different lowercase letters on the bars indicate significant differences caused by different storage times (P < 0.05). *The orders of lowercase letters are for AP, VP, and MAP, respectively.

Changes in protein solubility are a key indicator of protein denaturation, influenced by the balance between hydrophilicity and hydrophobicity during storage (Kaewthong et al., 2019). Water-soluble protein, referred to as sarcoplasmic protein, gradually decreased from 2.52 to 1.37–1.64% by Day 12 (P < 0.05) (Fig. 1A). This could reflect the denaturation of proteins (i.e., creatine kinase, myoglobin, and other enzymes) during storage. This leaching of water-soluble proteins may contribute to a pale, soft, exudative (**PSE**) appearance in the meat, as noted by Singh and Deshpande (2018) AP samples showed the greatest loss of water-soluble protein compared to VP and MAP at both Day 6 and Day 12 (P < 0.05), which was plausibly responsible for the accelerated development of undesirable characteristics during storage. The low levels or absence of O_2_ in VP and MAP packaging may slow enzymatic hydrolysis and protein denaturation, potentially caused by microorganisms. Moreover, increased weight loss during extended storage could be linked to greater water-soluble protein loss, which was more pronounced in the AP condition. Salt-soluble protein, which represents myofibrillar protein, makes up over 50% of total protein and forms a three-dimensional gel network that influences the WHC and texture of meat (Ji et al., 2021). In KC meat, salt-soluble protein increased from 6.50 to 11.12% by Day 12 (Fig. 1B). As storage time progresses, proteolytic enzymes degrade myofibrillar protein structures, weakening protein–protein interactions and increasing protein solubility (Feng et al., 2020; Pongsetkul et al., 2022a). This proteolysis promotes partial protein unfolding, exposing both hydrophilic and hydrophobic groups, which enhances extractability in salt solutions and contributes to meat tenderization (Koohmaraie et al., 2002). Notably, no significant differences in salt-soluble protein were observed among the different packaging treatments at any storage period (P > 0.05), indicating a comparable extent of myofibrillar protein unfolding regardless of packaging conditions. The relatively constant salt-soluble protein levels across packaging treatments may be attributed to gradual unfolding accompanied by a greater exposure of hydrophobic than hydrophilic groups, which limits further increases in solubility, as previously reported by Maity et al. (2012).

#### Total and insoluble collagen

Collagen, the intramuscular connective tissue surrounding muscle fibers, plays a role in meat texture, with its solubility and crosslinks being positively associated with sensory tenderness (Li et al., 2022). No significant difference in total collagen (0.36–0.43%) was observed among samples over 12 days of storage (P > 0.05) (Fig. 1C), consistent with the findings by Zhu et al. (2023) on broiler meat during 3 days of cold storage. Jeon et al. (2010) suggested that the inherent stability of collagen in meat was due to the same genotypes and age at slaughter. Interestingly, total collagen in KC was higher than in Ligor hybrid chicken (Thai crossbred) (0.14–0.21%) and Korean native chicken (*woorimotdak*) (0.2–0.3%) (Jeon et al., 2010; Panpipat et al., 2022). Nevertheless, all samples showed an increase in insoluble collagen at prolonged storage (Fig. 1D), highest in AP and lowest in VP (P < 0.05). Higher insoluble collagen contributes to muscle stiffness and reduced fleshiness (Zhu et al., 2023). Collagen crosslinks, influenced by meat age, processing, and storage, lead to increased insoluble collagen and reduced protein functionality (Katemala et al., 2022; Xing et al., 2021). Protein oxidation forms collagen crosslinks (i.e., pyridinoline), stabilizing collagen and making it more resistant to breakdown and less soluble during storage, which contributes to increased meat stiffness (Coró et al., 2002). Besides, Lund et al. (2007) reported that higher O_2_ levels in AP and MAP promoted protein oxidation, facilitating crosslink formation (i.e., crosslinked myosin heavy chain), while VP (with no O_2_) prevented this. Thus, higher O_2_ in AP could facilitate protein oxidation, modifying collagen’s functional amino groups and affecting its solubility more than VP and MAP.

#### Protein oxidation

Protein oxidation, driven by carbonylation of amino acid side chains (i.e., lysine, threonine, arginine, and proline), leads to denaturation and loss of myofibrillar protein functionality (Soncu, 2020). Initially, oxidation increases carbonyl content, followed by a depletion of thiols over storage (Estévez and Xiong, 2021). During 12 days of storage, protein carbonyl content increased among samples (from 0.75 to 4.45 nmol/mg protein) at varying rates (Fig. 1E), indicating that oxidation progressed differently depending on the packaging conditions. AP contained higher carbonyl levels than VP and MAP within the same day of storage (P < 0.05), as the presence of O_2_ facilitated metal ion-catalyzed oxidation. This crosslink formation from the oxidation process impaired WHC, implicating changes in meat texture. However, VP and MAP exhibited similar carbonyl formation rates throughout storage (P > 0.05), suggesting that reduced O_2_ slowed oxidation. This effect corresponded well with a lower loss of free thiols in VP and MAP samples compared to AP (P < 0.05) (Fig. 1F), which was consistent with the findings by Jongberg et al. (2014) in their study on broiler meat. Free thiols (R-SH) enhance protein polymerization or oxidation-mediated protein modification in meat, while their loss indicates the formation of disulfide crosslinks (Jongberg et al., 2014). Thiol content in AP samples dropped the fastest, from 38.92 to 30.02 nmol/mg protein, indicating oxidation of thiol groups and disulfide crosslinking due to O_2_ exposure, which led to protein denaturation and aggregation, typically resulting in a stiffer, more rigid meat texture (Soncu, 2020). Therefore, AP showed more pronounced protein oxidation, while reduced O_2_ packaging (VP and MAP) mitigated oxidation and preserved meat texture.

#### SEM image

**Fig. 2:**
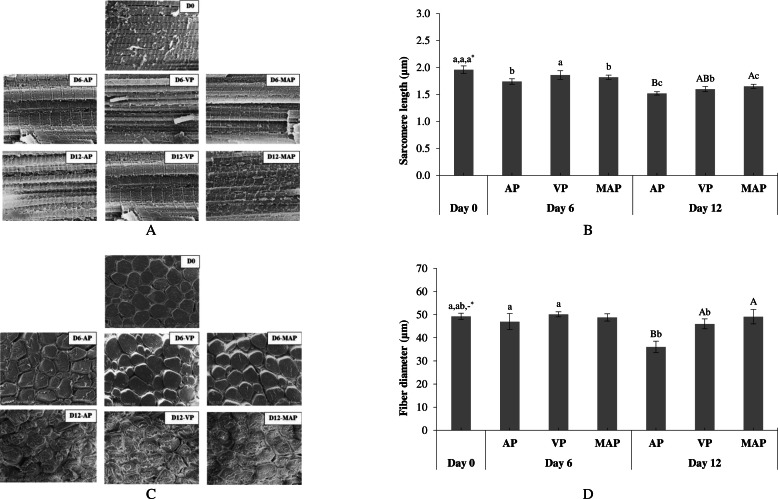
SEM images of longitudinal (A) sarcomere length (B), SEM images of transverse sections (C), and fiber diameter (D) of KC meat stored under various packaging conditions at 4°C for 12 days. Different uppercase letters on the bars indicate significant differences caused by different packaging conditions at the same storage time (P < 0.05). Different lowercase letters on the bars indicate significant differences caused by different storage times (P < 0.05). *The orders of lowercase letters are for AP, VP, and MAP, respectively.

The microstructural changes in KC meat during cold storage under different packaging conditions are shown in Fig. 2**.** Based on the longitudinal section (Fig. 2A), sarcomere shrinkage was observed over time, with sarcomere length decreasing from Day 0 (1.96 μm) to Day 12 (1.52–1.65 μm) (Fig. 2B). On Day 12, AP had the shortest sarcomere length (P < 0.05), while VP and MAP did not differ, indicating greater myofibrillar protein shrinkage in AP, particularly after prolonged storage for more than 6 days. Nawaz et al. (2022) proposed that sarcomere contraction, caused by increased overlap of actin and myosin filaments, may lead to disulfide bond formation, resulting in tougher meat. Shrinkage of myofibrillar proteins is associated with protein oxidation, which promotes crosslinking and disulfide bond formation, mechanically constricting the muscle and reducing WHC, leading to decreased meat juiciness (Tang et al., 2020). Protein carbonylation further contributes to myofibrillar aggregation by unfolding proteins and exposing hydrophobic regions (Pongsetkul et al., 2024). The greater shrinkage in AP samples corresponded with higher protein degradation and oxidation (Fig. 1), explaining the faster development of undesirable toughness under normal air conditions.

Myofibrillar shrinkage during prolonged storage can also be observed in the transverse section (Fig. 2C). A reduced fiber diameter and increased gap between muscle fibers were noted as storage time progressed, suggesting myofibrillar shrinkage and collagen degradation, respectively. Larger gaps indicated collagen breakdown, which can lead to more tender meat, while reduced fiber diameter may suggest protein oxidation-driven aggregation. This phenomenon was most pronounced in AP samples, as confirmed by the lowest fiber diameter on Day 12 (Fig. 2D). Although VP and MAP, which were absent of O_2,_ slowed protein oxidation (Fig. 1D and 1E), enzymatic activities (i.e., proteolysis) could still affect muscle proteins, leading to sarcomere width (myofibril diameter) shrinkage over storage (Vaskoska et al., 2020). Proteolytic enzymes may also degrade collagen around muscle fibers, but to a lesser extent than in AP samples. Overall, these findings confirm that AP caused greater myofibril disruption, which could lead to a lower WHC and tougher texture, compared to VP and MAP.

#### FTIR spectra

**Fig. 3:**
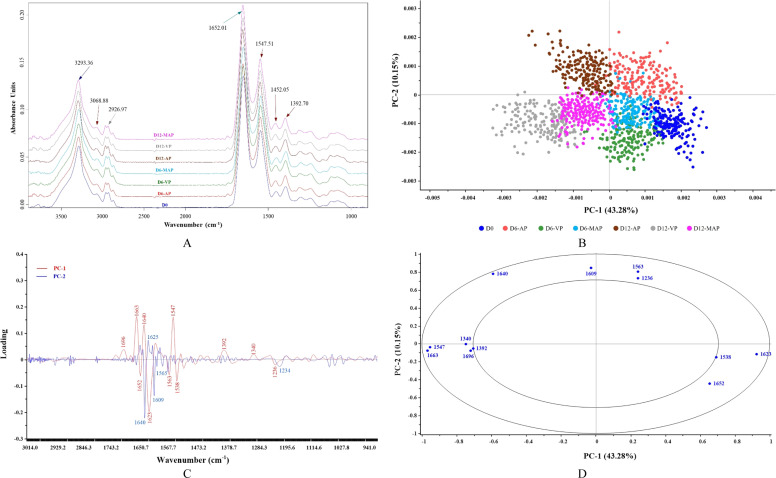
Average original SR-FTIR spectra (A), PCA score plot (B), loading plot (C), and correlation loading plot (D) of KC meat stored under various packaging conditions at 4°C for 12 days. The obtained spectra were analyzed in the regions of 3,000–2,800 cm^−1^ and 1,800–900 cm^−1^.

The original FTIR spectra of AP, VP, and MAP samples over 12 days of cold storage revealed various biomolecules and their corresponding functional groups (Fig. 3A). The identified spectra were of lipids (3000–2800 cm^−1^), amide I (1700–1600 cm^−1^), amide II (1600–1500 cm^−1^), amide III (1340 cm^−1^), and carbohydrates/glycogen (1250–900 cm^−1^). All samples displayed similar spectra with seven dominant peaks of varying intensities. The N–H stretching vibration peak at 3293 cm^−1^ indicates H-bond involvement in peptides (Ahmad et al., 2019). A lower intensity of this peak correlates with greater collagen peptide interaction via H-bonds (Indriani et al., 2023), suggesting increased intensity during storage may indicate H-bond loss due to protein denaturation or oxidation. Peaks at 3069 cm^−1^ and 2927 cm^−1^ represent asymmetric stretching vibrations of –NH_3_^+^ and =CH in lipids, while amide I and II were identified at 1652 cm^−1^ and 1548 cm^−1^, respectively, along with the bending of CH_2_ and CH_3_ at 1425 cm^−1^ and 1393 cm^−1^. Amide I relates to C=O bond stretching, forming H-bonds between adjacent chains, while amide II involves N–H deformation and C–N elongation (Pongsetkul et al., 2024). These functional groups are crucial for investigating conformational changes in protein secondary structures due to external factors (i.e., pH shifts, temperature, and/or hydration) (Nian et al., 2019).

**Fig. 4:**
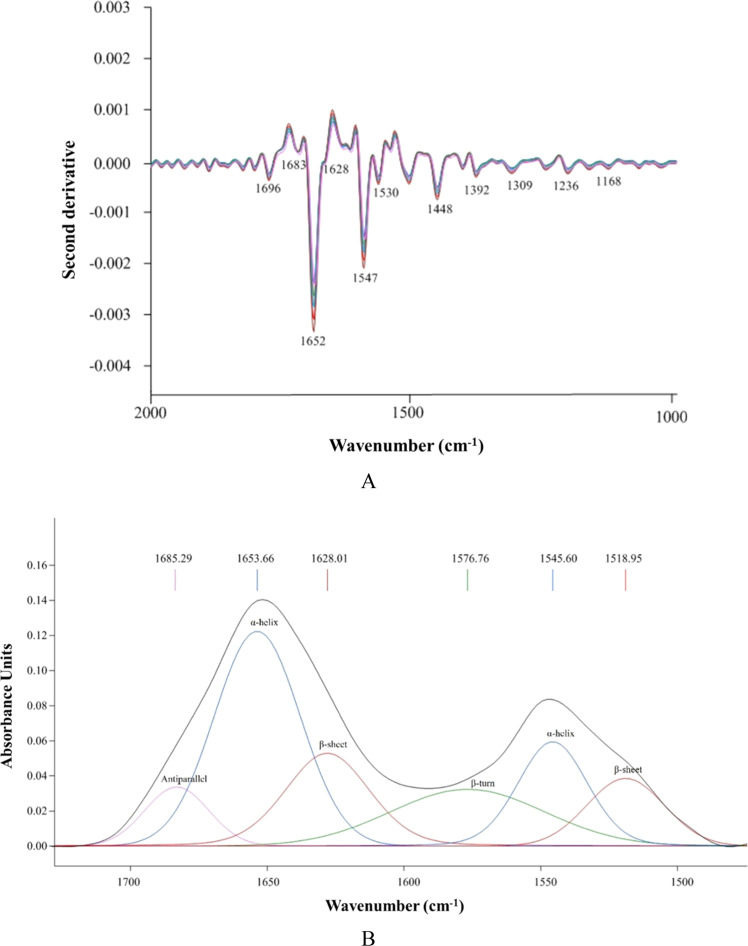
Second derivative-transformed spectra (A) and the peak deconvolution of amide I and secondary structure protein band assignment (B) of KC meat stored under various packaging conditions at 4°C for 12 days. Original spectra are shown black, fitted spectra in colors. The individual bands are illustrated in different colors respective to their secondary structures of protein.

The biomolecule spectra (3000–2800 cm^−1^ and 1800–900 cm^−1^) were selected for further correlation plotting (Fig. 3B, C, and 3D). The score plot, showing 53.43% total variability (Fig. 3B), indicated that PC-1 (43.28%) differentiated the samples based on storage time, with a progressive clustering observed moving from the right to the left as storage time increased. In contrast, PC-2 (10.15%) separated AP, which clustered in the upper part, from VP and MAP, found in the lower part. This indicated that storage time has a greater impact on biomolecule spectra changes compared to packaging conditions. VP and MAP exhibited similar spectral changes due to their close clustering, remaining distinct from AP throughout storage. The absence of O₂ in VP and MAP highlights its role in regulating biomolecular changes that affect meat quality, particularly texture. Additionally, the loading plot (Fig. 3C) and correlation loading plot (Fig. 3D) identified twelve (12) peaks in sample differentiation (P < 0.05). Notably, peaks at 1652 cm^−1^, 1623 cm^−1^, and 1538 cm^−1^, representing amide I and II, significantly differentiated on Day 0 from other samples (P < 0.05), as they were located at the same point in the plotting area (the lower right side of the graph). This demonstrated the specific integrity of the protein’s secondary structure in fresh KC meat. Peak shifts and intensity changes in amide I and II during storage reflect protein structural changes and quality deterioration (Vasconcelos et al., 2014). A shift to lower wavenumbers in amide I suggests H-bond loss or conformation changes, likely due to denaturation, while a shift to higher wavenumbers may indicate increased H-bonding or structural stabilization, although this is less common in stored meat (Bai et al., 2023). In this study, peak shifts of amide I and II were observed, with extended storage time having a more significant impact than the packaging conditions. Peaks at 1563 cm^−1^ and 1236 cm^−1^ distinguished on Day 6, correlating with amide II (*α*-helix) and carbohydrates or glycogen, respectively, suggesting protein denaturation caused by enzymatic or biochemical reactions. By Day 12, the secondary protein structure was characterized by spectra at 1696cm^−1^, 1663 cm^−1^, 1640 cm^−1^, 1609 cm^−1^, 1392 cm^−1^, and 1340 cm^−1^ (P < 0.05) (Fig. 3D). Notably, the decrease in the 1340 cm^−1^ peak, corresponding to amide III, suggests a transition from disordered α-helical structure to random coils, indicating a reduction in collagen’s structural integrity (Indriani et al., 2022). The alteration of this peak with increased storage time, particularly on Day 12, therefore, corresponded with changes in total and insoluble collagen contents (Fig. 1C and D), as well as the development of a larger gap among muscle fibers, as depicted in the SEM microstructural changes (Fig. 2). Based on the variations in peak shifts and intensities of amide I and II among samples, which were associated with the changes in secondary protein structure (1700–1500 cm^−1^), these areas were then selected for secondary derivatization and curve fitting, as shown in Fig. 4A and Fig. 4B, respectively.

The relative content of protein secondary structures obtained through curve fitting is reported in Table 1. Notably, *α*-helix and *β*-sheet comprised over 80% of the protein secondary structures, followed by antiparallel and *β*-turn across all samples during storage. The *α*-helix content decreased from 50.16% on Day 0 to 37.04% on Day 12 for AP (P < 0.05). According to Bai et al. (2023), this loss reduces protein rigidity, leading to a less firm texture, while Nawaz et al. (2022) noted that it also decreases water retention, increasing drip loss and making the meat drier. Additionally, a reduction in *α*-helix decreases muscle protein elasticity, making the meat more fragile or less chewy, depending on the extent of denaturation (Roobab et al., 2024). The exposed proteins are more susceptible to proteolysis, softening the meat and potentially resulting in undesirable mushiness (Ahmed et al., 2015; Lund et al., 2007). AP exhibited the greatest loss of α-helix, indicating the fastest protein denaturation during storage, which resulted in the most pronounced loss of structural integrity and water retention. In addition, a greater conversion of *α*-helix to antiparallel *β*-sheet structures was observed in AP during prolonged storage, leading to increased shear force and hardness of KC meat. During this structural transition, hydrogen bonds stabilizing the *α*-helix were disrupted, thereby increasing surface hydrophobicity as antiparallel *β*-sheets aggregated (Li et al., 2018). Consequently, the formation of tightly-packed and less flexible antiparallel *β*-sheet structures contributed to higher shear force and hardness values (Roeters et al., 2017). These structural changes could result in undesirable textural changes, making the meat drier and tougher upon extensive cooking, thereby negatively affecting sensory acceptance. In contrast, VP and MAP showed a lower rate of *α*-helix loss (P < 0.05), suggesting better meat integrity and preservation of texture in reduced O_2_ conditions compared to AP. An increase in *β*-sheet content was observed as storage time extended (P < 0.05). While *β*-sheet contributes to firmness and rigidity, excessive formation can lead to protein aggregation and toughness (Nian et al., 2019). The concurrent decrease in *α*-helix and increase in *β*-sheet further confirmed the disruption of protein structure, contributing to undesirable textures, such as toughness and chewiness. These structural changes could also reduce meat digestibility (Bai et al., 2023). Guo et al. (2019) noted that the reduction of *α*-helix and increase of *β*-sheet led to decreased hardness and springiness, alongside an increase in gumminess in ripened chicken breast meat. Similar to *α*-helix, *β*-turn showed a decreasing trend as the storage period extended (P < 0.05). *β*-Turn plays a role in protein folding and stability, and its high or low content contributed to meat tenderness or toughness, respectively (Pongsetkul et al., 2024). AP showed a greater loss of *β*-turn compared to VP and MAP at the same storage (P < 0.05), which aligned well with the greater protein denaturation and oxidation, hindering water retention in meat packed under original atmosphere conditions. The proportion of antiparallel structures increased from 9.61 to 13.45–14.85% after 12 days of storage for AP (P < 0.05). However, no significant differences among packaging conditions were observed at Day 12 (P > 0.05). This increase contributes to protein aggregation and crosslinking (Sun et al., 2018). The rise in antiparallel structures over storage time may indicate amino acid oxidation, leading to *α*-helix modification and disruption of H-bonds, which help preserve the helical structure. This was particularly relevant for KC meat exposed to O_2_ during extended storage, particularly under normal atmospheric conditions.

**Table 1:** Relative content (%) of protein secondary structures obtained from amide I spectral profile (after curve fitting) of KC meat stored under various packaging conditions at 4°C for 12 days.

Sample	Protein secondary structure			
	*α*-helix	*β*-sheet	*β*-turn	Antiparallel
*Day 0*	50.16 ± 1.01^a,a,a*^	36.12 ± 0.98^c,b,b*^	4.11 ± 0.17^a,a,a*^	9.61 ± 0.93^b,b,b*^
*Day 6*				
AP	46.39 ± 1.02^ABb^	39.66 ± 1.50^b^	3.36 ± 0.11^Bb^	10.59 ± 0.97^Ab^
VP	48.06 ± 0.92^Ab^	40.35 ± 1.61^a^	3.60 ± 0.18^ABb^	7.99 ± 0.42^Bc^
MAP	46.05 ± 0.61^Bb^	41.12 ± 2.03^a^	3.88 ± 0.25^Aa^	8.95 ± 0.89^ABb^
*Day 12*				
AP	37.04 ± 1.44^Bc^	46.22 ± 2.56^Aa^	2.78 ± 0.29^Bc^	13.96 ± 1.06^a^
VP	42.17 ± 2.05^Ac^	39.58 ± 2.01^Ba^	3.40 ± 0.18^Ab^	14.85 ± 1.12^a^
MAP	41.88 ± 1.13^Ac^	40.93 ± 1.04^Ba^	3.74 ± 0.20^Aa^	13.45 ± 0.63^a^

Data are expressed as mean ± SD (*n* = 3).

Different uppercase letters in the same column indicate significant differences caused by different packaging conditions at the same storage time (P < 0.05). Different lowercase letters in the same column indicate significant differences caused by different storage times (P < 0.05). *The orders of lowercase letters are for AP, VP, and MAP, respectively.

### Changes in textural characteristics of KC meat during storage

#### pH

During 12 days of storage, pH decreased from 6.09 to 5.79 for all packaging conditions (P < 0.05) (Table 2). AP exhibited a gradual pH decline, while VP and MAP maintained a stable pH from Day 6 to Day 12 (P < 0.05), suggesting faster spoilage in AP. The presence of O_2_ in AP likely promoted the growth of aerobic spoilage bacteria that produce organic acids, lowering the pH. Indriani et al. (2025) noted that microbial diversity and dynamics influence pH changes, with anaerobic bacteria favored by the absence of air in VP and the presence of CO₂ in MAP producing compounds such as lactic acid, which affect pH, flavor, and odor. These findings explained why VP and MAP showed a pH drop during the early storage period, likely due to anaerobic bacterial activity, while their pH remained stable during extended storage, possibly due to a balance of compounds that regulate the pH. Wen et al. (2020) linked bacterial degradation of proteins to NH_3_, amines, and organic sulfide release, contributing to pH regulation during extended storage. Bao and Ertbjerg (2019) further highlighted the role of O_2_, in protein modification, affecting pH. Yang et al. (2022) postulated that, after glucose depletion, spoilage bacteria preferentially metabolize nitrogenous amino acids, potentially leading to a subsequent increase in pH. In addition, pH changes can alter the net charge and solubility of muscle proteins, thereby influencing protein structure and their spatial arrangement within the muscle matrix (Bao and Ertbjerg, 2019). Typically, a pH shift away from the isoelectric point (**pI**) (5.0–5.5 for chicken) improves WHC (Zhao et al., 2017). The greater pH reduction in AP, therefore, corresponded to lower water retention, as indicated by the lowest WHC (Table 2) during prolonged storage, compared to VP and MAP.

**Table 2:** Physicochemical and textural characteristics, along with sensory liking score of KC meat stored under various packaging conditions at 4°C for 12 days.

Parameters	Day 0	Day 6			Day 12		
		AP	VP	MAP	AP	VP	MAP
*Physicochemical characteristics*							
pH	6.09 ± 0.05^a,a,a*^	5.93 ± 0.08^b^	5.86 ± 0.06^b^	5.84 ± 0.04^b^	5.79 ± 0.04^c^	5.82 ± 0.04^b^	5.86 ± 0.09^b^
WHC (%)	72.43 ± 0.62^a,a,a*^	69.46 ± 1.03^Bb^	72.05 ± 1.20^Aa^	71.98 ± 0.60^Aa^	64.80 ± 1.03^Bc^	68.02 ± 0.59^Ab^	70.16 ± 0.41^Ab^
Weight loss (%)	-	3.55 ± 0.29^Ab^	2.01 ± 0.40^Bb^	2.20 ± 0.30^Bb^	6.12 ± 0.51^Aa^	4.50 ± 0.46^Ba^	3.53 ± 0.40^Ca^
Cooking loss (%)	16.95 ± 0.89^c,ab,b*^	19.43 ± 1.03^Ab^	16.12 ± 1.15^Bb^	16.90 ± 0.88^Bb^	22.01 ± 1.06^Aa^	18.26 ± 0.90^Ba^	18.53 ± 0.59^Ba^
*Textural characteristics*							
Shear force (N)	22.62 ± 0.54^b,b,b*^	22.03 ± 1.05^b^	23.02 ± 0.42^b^	22.96 ± 0.60^ab^	27.95 ± 0.60^Aa^	24.62 ± 0.42^Ba^	23.59 ± 0.36^Ca^
Hardness (N)	59.19 ± 1.59^c,b,a*^	65.88 ± 2.00^Ab^	61.13 ± 2.12^Bb^	59.28 ± 3.38^Ba^	78.26 ± 2.60^Aa^	68.22 ± 2.04^Ba^	62.43 ± 2.45^Ca^
Chewiness (N*mm)	32.66 ± 1.51^b,b,b*^	34.61 ± 0.48^Ab^	32.06 ± 1.03^Bb^	31.99 ± 1.55^Bb^	41.28 ± 1.06^Aa^	37.50 ± 2.00^Ba^	35.99 ± 0.90^Ba^
Springiness (mm)	4.46 ± 0.29	4.22 ± 0.49	4.98 ± 0.30	4.98 ± 0.41	5.16 ± 0.61	4.98 ± 0.40	5.02 ± 0.41
Cohesiveness	0.18 ± 0.01	0.18 ± 0.03	0.18 ± 0.02	0.16 ± 0.04	0.13 ± 0.02	0.16 ± 0.03	0.16 ± 0.03
*Sensory liking score (9-point hedonic scale)*							
Tenderness	7.30 ± 0.33^a,a,a*^	6.38 ± 0.32^b^	6.90 ± 0.40^a^	6.34 ± 0.30^b^	5.15 ± 0.42^Bc^	5.91 ± 0.35^Bb^	6.62 ± 0.33^Ab^
Juiciness	8.01 ± 0.51^a,a,a*^	6.13 ± 0.50^Bb^	7.05 ± 0.36^Ab^	7.10 ± 0.29^Ab^	5.49 ± 0.33^Bb^	6.02 ± 0.40^Ac^	6.62 ± 0.40^Ab^
Overall texture	7.95 ± 0.38^a,a,a*^	5.80 ± 0.31^Bb^	6.80 ± 0.29^Ab^	6.80 ± 0.51^Ab^	5.02 ± 0.38^Bc^	6.45 ± 0.61^Ab^	6.32 ± 0.30^Ab^

Data are expressed as mean ± SD (*n* = 3).

Different uppercase letters in the same row indicate significant differences caused by different packaging conditions at the same storage time (P < 0.05). Different lowercase letters in the same row indicate significant differences caused by different storage times (P < 0.05). *The orders of lowercase letters are for AP, VP, and MAP, respectively.

#### WHC, weight loss, and cooking loss

A decrease in WHC was observed in all samples during extended storage (P < 0.05) (Table 2). This decline may be attributed to crosslink formation, stabilizing protein aggregates, and leading to myofibril shrinkage and mechanical constriction, which limit swelling (Bao and Ertbjerg, 2019). AP possessed the fastest WHC decline (from 72.43 to 64.80%), consistent with its greatest reduction in weight loss and cooking loss (P < 0.05). Since WHC directly impacts weight and cooking loss, which affect meat yield and texture, it is critical for both profitability and meat quality. WHC involves a complex understanding associated with the composition and structure of muscle tissue (Katemala et al., 2022). Bowker and Zhuang (2015) noted that the denaturation governed the shift of proteins from the sarcoplasmic to the myofibrillar protein fraction, which could account for WHC in chicken breast meat. During storage, oxidative modifications cause protein polymerization and aggregation, resulting in deleterious effects in meats (Jongberg et al., 2014; Lund et al., 2007). Interestingly, on Day 12, VP and MAP showed a comparable low reduction in WHC (68.02% and 70.16%, respectively) and weight loss (4.50% and 3.53%, respectively) (P < 0.05), suggesting that VP and MAP better preserved yield and desirable texture than AP. The CO₂ in MAP could stabilize protein structures during storage, improving water retention compared to normal or absent air conditions. High CO_2_ levels were expected to preserve WHC more effectively than high O_2_ levels. By limiting O_2_ exposure, both VP and MAP help maintain functional properties, particularly WHC, weight loss, and cooking loss, with MAP offering superior preservation, likely due to its higher CO₂ content. Hauschild et al. (2021) reported that increasing CO₂ relative to O₂ in packaging enhances WHC preservation due to its more active role in inhibiting microbial growth.

#### Shear force and TPA

According to Table 2, all samples demonstrated an increase in shear force from Day 6 to Day 12, corresponding with a rise in insoluble collagen content (Fig. 1D). This suggested that collagen degradation or transformation contributed to a tougher texture during extended storage. The increase in shear force is linked to the formation of pyridinoline crosslinks in oxidized collagen due to O_2_ exposure or enzyme activities (Li et al., 2022). AP had the highest shear force, which aligned with its highest protein oxidation (carbonyl content, Fig. 1E) at 12 days of storage. In contrast, VP and MAP maintained tenderness, showing only a slight increase in shear force compared to Day 0. These results align with Nauman et al. (2022), who reported that broiler fillets stored under VP and MAP showed slightly higher shear force while still maintaining tenderness up to Day 10. Similarly, hardness increased over time in all samples, suggesting that KC meat became tougher (P < 0.05). Protein denaturation and oxidation-induced crosslinking likely contributed to this hardening effect (Purslow, 2005). Here, both VP and MAP positively impacted texture by promoting natural tenderization during cold storage. Notably, MAP had the lowest shear force (23.59 N) and hardness (62.43 N) on Day 12 (P < 0.05). This might be due to the protective effects of CO_2_ and N_2_ in MAP, which help retard oxidative changes and microbial activities (Gurunathan et al., 2022; Nauman et al., 2022). Couvert et al. (2023) reported that the minimum inhibitory concentration (**MIC**) of CO₂ for general bacterial growth ranged from 10 to 50%, whereas in the absence of CO₂, bacterial growth was favored even at low storage temperatures (8°C). Beyond its antimicrobial effect, CO₂, as a non-polar molecule, can induce molecular changes in muscle proteins by interacting with hydrophobic amino acid residues, thereby increasing hydrophobic exposure between protein molecules, extracting water, and altering water-protein interactions. These effects may promote protein conformational changes. Under pressures of up to 200 MPa, CO₂ has been shown to induce reversible alterations in protein secondary structure, characterized by a conversion of *α*-helix to *β*-sheet, *β*-turn, and random coil structures, which in turn influences meat texture (Guo et al., 2017). Consequently, the presence of CO₂ in MAP during cold storage provides superior microbial inhibition compared with air-free conditions, particularly during extended storage, while also contributing to improved texture preservation. Chewiness, which reflects the force required to fracture muscle fibers and fat while leaching water and influencing eating quality (Pongsetkul et al., 2024), increased the most in AP, rising from 32.66 to 41.28 N*mm (P < 0.05), making it less desirable. Springiness, which indicates how well the meat returns to its original form during masticating (Aguirre et al., 2018), showed no significant differences among samples (P > 0.05), suggesting a similar deformation profile in KC meat. Cohesiveness remained consistent across samples throughout storage (P > 0.05). This may indicate accelerated deformation of the structural integrity of KC meat over time, resulting in a less cohesive texture, particularly when stored in normal air. Overall, VP and MAP helped preserve texture quality, with MAP better retaining water and promoting tenderness compared to VP, leading to improved yield and meat quality.

### Sensory scores of KC meat during storage

Tenderness and juiciness are critical factors in meat quality, both associated with textural characteristics. Tenderness refers to the ease of chewing, while juiciness describes the amount of juice present in the mouth after five chews (Lund et al., 2007). During storage, a decline in these attributes’ scores (Table 2) indicated reduced texture quality in KC meat. AP consistently scored lower in juiciness and overall texture compared to VP and MAP at prolonged storage (P < 0.05), suggesting that vacuum and modified packaging effectively preserve the texture of KC meat, consistent with findings by Jongberg et al. (2014) and Nauman et al. (2022) in broilers. Notably, on Day 12, MAP achieved the highest tenderness score, which correlated with its lower shear force and hardness compared to others (P < 0.05). Nevertheless, both VP and MAP had similar juiciness and overall texture scores (P > 0.05), despite MAP demonstrating better WHC and moisture retention. The similarity in juiciness may be due to comparable springiness and cohesiveness between VP and MAP. Similarly, Nauman et al. (2022) reported that tenderness and juiciness in VP and MAP chicken breast remained acceptable up to Day 6, with no significant differences in the liking scores. Lund et al. (2007) reported that the reduced tenderness and juiciness during extended storage could be caused by the rapid crosslinking of myosin heavy chains via disulfide bonding under high O_2_ exposure. AP showed the sharpest decline in overall liking scores throughout storage (P < 0.05), while VP and MAP shared similar scores. These results suggested that limiting O_2_ exposure in KC meat packaging effectively mitigated texture deterioration as perceived by the panelists.

### PCA

**Fig. 5:**
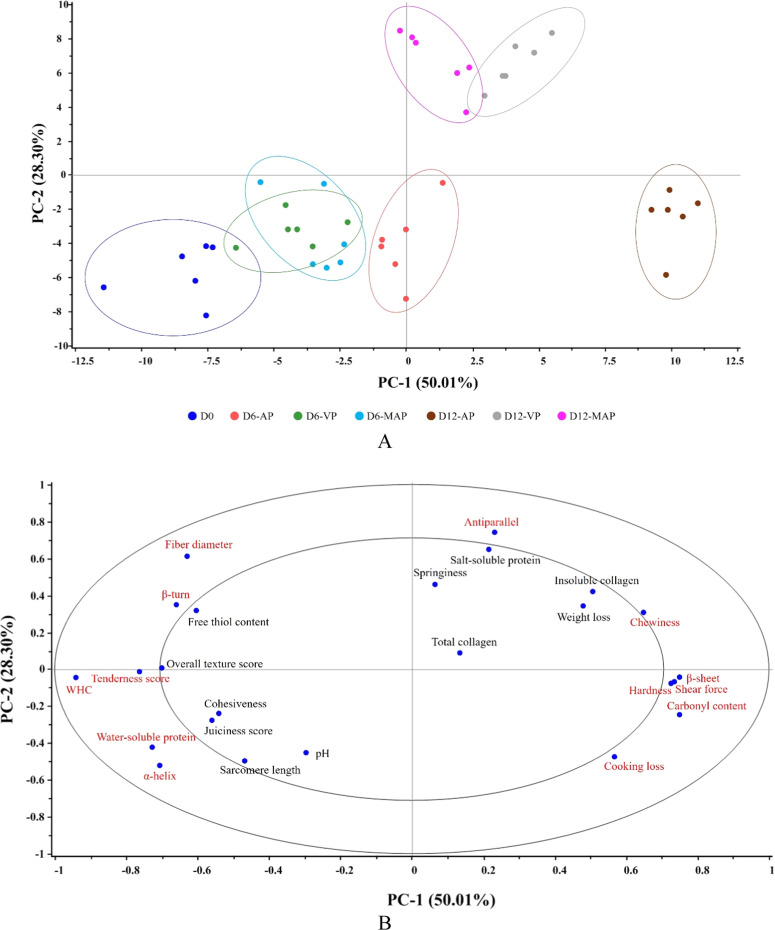
PCA score plot (A) and correlation loading plot (PC-1 vs. PC-2) (B) at 78.31% total variance of texture characteristics, secondary structure protein, and sensory scores of KC meat stored under various packaging conditions at 4°C for 12 days. Red letters indicate a significant level of P < 0.05.

The correlation between textural characteristics, secondary protein structure, and sensory scores of KC meat stored under various packaging conditions for 12 days was evaluated using PCA, explaining 78.31% of the total variance (Fig. 5). The PCA score plot demonstrated that PC-1 (50.01%) discriminated the samples by storage duration, with Day 0 and Day 6 clustered on the left side and Day 12 on the right side. Meanwhile, PC-2 (28.30%) distinguished AP from VP and MAP, located at the lower and upper sides, respectively (Fig. 5A). Based on the correlation loading plot (Fig. 5B), a strong correlation among *β*-sheet, shear force, hardness, and carbonyl content was observed, all of which were positively correlated with AP on Day 12 (P < 0.05). AP possessed greater oxidative protein modification, leading to structural changes through myofibrillar and collagen denaturation, as evidenced by a reduction in *α*-helix and the subsequent formation of non-helical structures, including *β*-sheet and antiparallel (Table 1), indicating a more rigid muscle structure. This was consistent with the negative correlation between this sample and *α*-helix, *β*-turn, fiber diameter, WHC, and water-soluble protein, which appeared on the opposite side of the plot (P < 0.05). Our results aligned with Beattie et al. (2004) and Katemala et al. (2021), who linked an increased *α*-helix to *β*-sheet ratio to a higher proportion of hydrophobic myofibrillar protein, contributing to higher shear force and hardness, thereby reducing tenderness and overall acceptability of meat during extended storage. Moreover, Li et al. (2022) suggested that protein oxidation, leading to pyridinoline crosslink formation, was associated with increased shear force. This reconfirms that protein oxidation accelerated muscle fiber shrinkage, reduced WHC, and caused moisture loss (including water-soluble proteins), ultimately leading to undesirable sensory attributes, such as a reduction in tenderness score. This description was consistent well with the lowest sensory scores observed for AP at Days 6 and 12 in this study (Table 2). Interestingly, antiparallel structures and chewiness distinguished VP and MAP from AP, showing a positive correlation (P < 0.05). It could be postulated that these reduced O_2_ conditions promoted a transition from helical to antiparallel *β*-sheets, rather than regular *β*-sheets. This suggested more orderly protein aggregates, which may have enhanced water retention and preserved meat firmness, particularly over extended storage periods, compared to normal packaging conditions. Antiparallel *β*-sheets, stabilized by more optimal H-bonding, are generally more rigid and structurally stable than parallel *β*-sheets, thereby contributing to the elasticity and chewiness observed in VP and MAP. Although parallel *β*-sheets also confer structural stability, their less optimal H-bonding renders them slightly less rigid (Xia et al., 2023). Moreover, antiparallel *β*-sheets possess more linear H-bonds and a more regular exposure of backbone polar groups, which facilitate the formation of structured hydration shells compared with regular *β*-sheets. Consequently, antiparallel *β*-sheets exhibit more stable hydration patterns, suggesting improved water retention in meat relative to regular *β*-sheet structures (Perets et al., 2020; Urbic and Dias, 2014). In addition to protein oxidation, proteolysis, primarily driven by bacterial activity, also alters meat texture by degrading myofibrils and connective tissues, leading to the softening of the meat. The breakdown of costameres, desmin, and other proteins (i.e., titin, nebulin, and tropomyosin) weakens muscle structure, reducing water retention and firmness, which results in a soft, mushy texture (Ahmed et al., 2015; Tang et al., 2020). As normal conditions promote greater microbial growth and proteolysis in meat than reduced O_2_ conditions (Nauman et al., 2022), this phenomenon corresponded to faster texture deterioration and lower texture acceptability of AP compared to VP and MAP. Therefore, VP and MAP preserved textural quality more effectively than AP during cold storage of KC meat. By containing no O_2_, these packaging methods not only slowed protein and lipid oxidation but also inhibited bacterial growth, thereby delaying meat decay. Nevertheless, MAP might be a better option for preserving KC meat due to its closer correlation to the Day 0 and Day 6 characteristics, compared to VP on Day 12. This option was reasonable for VP, as it could facilitate the growth of certain anaerobic bacteria, resulting in higher microbial populations that accelerate proteolysis and faster texture deterioration. Moreover, VP employed physical pressure to create vacuum conditions, resulting in a physical stress environment that may cause undesirable texture changes, such as increased water loss.

## Conclusion

Packaging conditions significantly influenced the molecular structure and textural changes of KC meat during storage. The presence of O₂ in AP accelerated protein degradation and oxidation, leading to structural transitions and undesirable textural attributes, including reduced WHC and tenderness. VP and MAP, which exclude O₂, effectively mitigated these negative textural changes, with MAP demonstrating superior preservation of texture quality, reflected in higher WHC, decreased weight loss/cooking loss, and improved tenderness scores, compared to VP, due to the presence of CO₂ in the packaging. PCA results revealed that storage time had a more substantial effect on textural changes than packaging conditions, with VP and MAP clustering closely together and distinctly separated from AP, especially on Day 12. Notably, the secondary protein structures associated with textural characteristics varied by packaging condition: *α*-helix in fresh meat (Day 0), *β*-sheet in AP, and antiparallel in VP and MAP. These changes corresponded well to a delay in decay when KC meat is preserved in reduced O₂ environments. Collectively, these findings highlight MAP as a particularly effective packaging strategy for maintaining the textural quality of KC meat during refrigerated storage, offering practical advantages for the poultry industry. Importantly, this study provides specific insights into slow-growing and crossbred chicken breeds such as KC, for which limited information is available, as most previous studies have focused primarily on fast-growing commercial broilers.

## Ethical approval

All procedures used in the present study were approved by the Ethics Committee on Animal Use and Human Research of the Suranaree University of Technology (SUT), Thailand (document ID: U1-02631-2559 and EC-67-122, respectively).

## CRediT authorship contribution statement

**Sylvia Indriani:** Writing – review & editing, Writing – original draft, Visualization, Investigation, Formal analysis. **Nattanan Srisakultiew:** Writing – review & editing, Visualization, Investigation, Formal analysis. **Soottawat Benjakul:** Writing – review & editing, Supervision, Methodology, Conceptualization. **Cynthia Andriani:** Writing – review & editing, Investigation, Formal analysis. **Kanjana Thumanu:** Writing – review & editing, Visualization, Supervision, Methodology, Data curation. **Passakorn Kingwascharapong:** Writing – review & editing, Supervision. **Samart Sai-ut:** Writing – review & editing, Supervision. **Jaksuma Pongsetkul:** Writing – review & editing, Resources, Project administration, Methodology, Funding acquisition, Data curation, Conceptualization.

## Disclosures

The authors declare that they have no known competing financial interests or personal relationships that could have appeared to influence the work reported in this paper.
